# Individual differences in envy experienced through perspective-taking involves functional connectivity of the superior frontal gyrus

**DOI:** 10.3758/s13415-020-00802-8

**Published:** 2020-06-18

**Authors:** Brennan McDonald, Kerstin Becker, Dar Meshi, Hauke R. Heekeren, Christian von Scheve

**Affiliations:** 1 Clinical Psychology and Behavioral Neuroscience, Faculty of Psychology, Technische Universität Dresden, Dresden, Germany; 2 Department of Political and Social Sciences, Institute of Sociology, Freie Universität Berlin, Berlin, Germany; 3 Department of Advertising and Public Relations, Michigan State University, East Lansing, MI USA; 4 Department of Education and Psychology, Biological Psychology and Cognitive Neuroscience Unit, Freie Universität Berlin, Berlin, Germany

**Keywords:** Social comparison, Envy, Emotion, fMRI, Functional connectivity, Superior frontal gyrus

## Abstract

Envy is the painful or resentful awareness of another’s advantage combined with a desire to possess that same advantage. Recent neuroscientific research has begun to shed light on the brain regions that process the experience of envy, including regions of the prefrontal cortex involved in emotional processing and social cognition. It is still unclear, however, which regions of the brain are functionally connected during the experience of envy. We recorded functional neuroimaging data while inducing simulated envy in participants, experienced through a perspective-taking hypothetical scenario task. In this task, participants took the perspective of a protagonist portrayed in a written description and compared themselves to either i) a self-similar/superior individual, ii) a self-dissimilar/superior individual, or iii) a self-dissimilar/average individual. During each comparison, participants also reported how much envy they experienced while taking the protagonists perspective. We demonstrate an inverse relationship in the connectivity of the left superior frontal gyrus to both the right supramarginal gyrus and the precuneus with respect to self-reported envy ratings across participants. In other words, we show that the greater the functional connectivity that the left superior frontal gyrus shares with the right supramarginal gyrus and precuneus, the less reported envy a participant experiences. Overall, our results are in line with previous research implicating the superior frontal gyrus in the reappraisal of negative emotions and extend these findings by showing this region is also involved in modulating the simulated experience of the social comparative, negative emotion of envy.

## Introduction

Social comparison involves individuals evaluating their own abilities and beliefs by comparing themselves to others (Festinger, 1954). When individuals engage in unfavorable upward social comparisons, they may experience the distressing emotion of envy (Salovey & Rodin, 1984; Silver & Sabini, 2006). Envy is defined as the painful or resentful awareness of another’s advantage combined with a desire to possess that same advantage (Merriam-Webster, 2018). Behavioral research on envy has established that the greater one’s tendency to compare oneself with others, the more dispositional envy one experiences (Smith, Parrott, Diener, Hoyle, & Kim, 1999; Zeelenberg & Pieters, 2007). Importantly, envy most often results from social comparisons with an individual who possesses the following two traits: i) a general similarity to the person engaging in the social comparison (e.g., age, sex, ethnic group, socioeconomic status, etc.), and ii) a key superior characteristic or possession that the person engaging in the social comparison lacks (e.g., status, high quality resources, access to mating opportunities, etc.) (Salovey & Rodin, 1984; Silver & Sabini, 2006). In other words, the envied person's superiority needs to be self-relevant to the person engaging in the social comparison both in trait similarity and the absence of a desired quality or resource to arouse a negative response. From an evolutionary perspective, envy prevents an individual from being outperformed by a direct competitor in a fitness-relevant domain: Envy motivates behaviors towards gaining a similar standing as a competitor or acting to remove a competitor’s advantage (Hill & Buss, 2006, 2008). Therefore, we experience envy when the positive attributes of another individual jeopardize our social standing (Crusius & Lange, 2016).

Along with the experience of an unpleasant emotional state, envy also is associated with a host of undesirable behaviors. These include hostility and aggression toward the envied person (Smith & Kim, 2007), a willingness to sacrifice a positive outcome to reduce the envied person’s advantage (Berke, 1988; Parks, Rumble, & Posey, 2002; Zizzo & Oswald, 2016), and the experience of schadenfreude (joy at another’s misfortune) toward the envied person’s suffering (van de Ven et al., 2015), even if unjustified (Zizzo & Oswald, 2016). Envy is further considered a central feature of narcissistic personalities (Krizan & Johar, 2012) and its presence is a diagnostic criterion for narcissistic personality disorder (Pincus & Lukowitsky, 2010). Life satisfaction also is lower in people who report experiencing envy often (Smith et al., 1999). Conversely, positive outcomes related to envy also have been reported, including motivating people to do better than their competitor (Protasi, 2016; van de Ven, Zeelenberg, & Pieters, 2009), by, for example, inspiring individuals to improve their position in the workplace (Schaubroeck & Lam, 2004).

Neuroimaging research has begun to shed light on the brain regions that process the experience of envy. Takahashi et al. (2009), using a protagonist-as-self (first person) perspective taking paradigm, found that the degree of simulated envy elicited by upwards social comparison was positively correlated with activity in the dorsal anterior cingulate cortex (ACC). In line with this result, a recent meta-analysis examining upward social comparison revealed consistent activation of the dorsal AAC and bilateral anterior insula across 44 comparison contrasts (Luo, Eickhoff, Hétu, & Feng, 2018). The dorsal ACC has been previously implicated in a range of functions, including reward evaluation, motivation, and conflict (Botvinick, Cohen, & Carter, 2004; Botvinick Todd S Braver et al., 2001; Heilbronner & Hayden, 2016; Shenhav, Cohen, & Botvinick, 2016), and is modulated by threat of self-concept (Moll, Zahn, De Oliveira-Souza, Krueger, & Grafman, 2005) and social pain (Eisenberger, 2012). The involvement of the dorsal ACC during the experience of envy has been substantiated by subsequent studies (Cikara & Fiske, 2013; Jankowski & Takahashi, 2014; Santamaría-García et al., 2017; Tanaka et al., 2019). An additional structure of note is the ventral striatum, a region strongly implicated in the processing of rewards (Haber, 2011), scenarios with a distribution of relative rewards (Bault, Joffily, Rustichini, & Coricelli, 2011; Dvash et al., 2010; Grygolec, Coricelli, & Rustichini, 2012; Kedia, Mussweiler, & Linden, 2014) and the processing of losses and gains for both the self and others (Delgado, Li, Schiller, & Phelps, 2008; Lieberman and Eisenberger, 2009; Zink et al., 2008). For example, Takahashi et al. (2009) found that the greater simulated schadenfreude reported from a protagonist-as-self perspective the greater the activation in the ventral striatum, whereas Dvash et al. (2010) demonstrated that the response of the ventral striatum is modulated by the degree to which a putative player experiences gains and losses relative to the participant, with losses producing self-reports of schadenfreude from the participant and increased ventral striatum activation and gains producing decreased ventral striatum activation and self-reports of envy.

Along with dorsal ACC and ventral striatum activation, other regions of the prefrontal cortex (PFC) have also been implicated in the processing of envy (Harris & Fiske, 2007; Santamaría-García et al., 2017; Shamay-Tsoory, Tibi-Elhanany, & Aharon-Peretz, 2007; Xiang, Kong, Wen, Wu, & Mo, 2016). These include the medial PFC (Harris & Fiske, 2007), a region reliably activated by social cognition tasks including perspective taking (Reniers et al., 2012; Van Overwalle, 2009), the ventromedial PFC (Shamay-Tsoory et al., 2007), implicated in the regulation and inhibition of emotional responses (Goldin, McRae, Ramel, & Gross, 2008), the dorsolateral PFC (Santamaría-García et al. 2017), and the middle and inferior frontal gyri (Xiang et al., 2016).

Finally, a recent study (Santamaría-García et al. 2017) using a lesion model to investigate envy substantiated the role of the ACC while also demonstrating a negative correlation between the reported experience of envy and gray matter in posterior regions of the cortex, including the angular gyrus, implicated previously in moral judgements (Moll et al., 2005; Raine & Yang, 2006) and representations of the self and others (Legrand & Ruby, 2009), and the precuneus, which has been associated with mentalizing abilities and social decision-making processes (Bzdok et al., 2012; Schlaffke et al., 2015). Taken together, these neuroimaging results implicate a series of brain regions in the experience of envy or the simulated experience of envy through perspective-taking; however, to the best of our knowledge, no study has yet revealed which regions of the brain are functionally connected during envy processing.

The goal of the current study, therefore, was to investigate the functional connectivity between brain regions during envy processing. We chose to use an above-mentioned, previously established paradigm in which participants experience envy through perspective-taking (Takahashi et al., 2009). We did this because of people’s strong tendency to underreport their personal feelings of envy (Habimana & Massé, 2000; Silver & Sabini, 2006), and evidence for brain structure differences underlying this social desirability bias (Andrejević, Meshi, van den Bos, & Heekeren, 2017). As such, for the current study we used the Takahashi et al. (2009) paradigm to create the possibility to report feelings of envy indirectly. We did this by asking our participants how envious they felt when placing themselves in a scenario, taking the perspective of the scenario’s protagonist (protagonist-as-self). We thus simulated subjective envy in participants by providing them with a first-person perspective-taking hypothetical scenario task (see *Procedure* section in *Materials and Methods*). Importantly, previous findings indicate that negative affective responses can be induced by taking another’s perspective (Todd, Forstmann, Burgmer, Brooks, & Galinsky, 2015; Gilead et al., 2016; Binyamin-Suissa et al., 2019; Takahashi et al., 2009). For example, Gilead et al. (2016) found that taking the perspective of either a tough/resilient or sensitive/squeamish individual could differentially simulate the expected negative affective state of the target.

With both the above literature and our chosen experimental task in mind, we hypothesized that simulating the negative emotion of envy would modulate brain regions involved in the experience of negative affect and emotional appraisal indicative of envy processing, including the ACC and medial prefrontal cortex. In addition, due to the nature of the experimental task we expected regions involved in perspective-taking and self/other processing to mediate the simulated experience of envy, including the angular gyrus and precuneus. With regard to functional connectivity, we hypothesized that the regions involved in envy would interact with regions implicated in self/other processing and evaluation, with the strength of functional connectivity correlating with individual differences in the experience of envy. To note, previous neuroimaging results with the protagonist-as-self paradigm that we employ below (Takahashi et al., 2009) could have been more robust—results were not corrected for multiple comparisons in their regions of interest and the extent of their minimum cluster size threshold was a mere five voxels. With this in mind, we conducted a rigorous and exploratory whole-brain analysis to reveal regions of the brain involved in envy processing. We then used these regions to conduct psychophysiological interaction (PPI) analyses. This therefore directly addressed our research question, revealing the functional brain network that lies at the core of envy processing, albeit in this simulated envy scenario.

## Materials and methods

### Participants

Twenty-three individuals (11 males) between 20 and 32 years of age (M = 27.2, standard deviation [SD] = 3.3) took part in the experiment. We based our sample size, similar to our experimental paradigm, on Takahashi et al. (2009), where the authors assayed 19 individuals. All participants were right-handed, with normal or corrected-to-normal vision, and reported no prior history of neurological or psychiatric disorder, with no current use of any psychoactive medications. Participants were recruited via flyer advertisements placed at Freie Universität Berlin, and all were native German speakers. Two participants were excluded from analysis, leaving a total of 21: one due to excessive head motion (>3 mm) during the functional magnetic resonance imaging (fMRI) procedure, and the other for not understanding the experiment instructions. The study was approved by a local ethics committee and conducted in accordance with the Declaration of Helsinki. Participants received 15€ for their participation and gave written informed consent before investigation.

### Procedure

Before the scanning procedure, each participant was presented with four scenarios in German. Each scenario described a person: either the protagonist (whose perspective the participant takes) or a target individual (3 others). These scenarios were developed and validated previously for an experiment on envy originally in Japanese by Takahashi et al. (2009) with an English translation provided with the publication (for the current study the German translation was derived from the English version). While reading the scenarios, participants were asked to identify with and take the perspective of the protagonist and to compare themselves with the three other persons described in the scenarios from this first-person perspective. The scenarios were divided into two sequential parts; the first part described the university life of the topic person on several highly self-relevant comparison dimensions: for example, academic achievement, achievements in sports, and degree of popularity. The second part of the scenarios described the post-university graduation life of the topic person, also with several highly self-relevant comparison domains: for example, performance in an important job interview, prestige of current workplace, and level of income. Because superiority and similarity of self-relevant comparison domains are necessary preconditions for inducing envy (Salovey & Rodin, 1984; Schaubroeck & Lam, 2004; Smith et al., 1999), superiority and similarity were varied experimentally to induce envy. This was achieved by describing the three comparison persons as either superior or mediocre with respect to the achievements of the protagonist and similar or dissimilar with respect to the interests and preferences of the protagonist. This resulted in three experimental conditions (Takahashi et al., 2009): (1) With regard to the protagonist, this comparison person possessed superior qualities and achievements and was similar to the protagonist with regard to shared interests, activities, and goals (SpHi = superior with high similarity). (2) The second comparison person possessed superior achievements compared to the protagonist but had dissimilar interests, activities, and goals (SpLo = superior with low similarity). (3) The third comparison person had average accomplishments, which were similar to the accomplishments described for the protagonist, and therefore, this person did not possess superior achievements in comparison to the protagonist. Furthermore, this person also had dissimilar interests, activities, and goals compared to the protagonist. As a result, the third person shared no relevant comparison domains with the protagonist (AvLo = average with low similarity). Scenarios were individualized according to gender and actual field of study of the participants (humanities, arts, or natural sciences) to further facilitate identification with the protagonist. For example, female participants studying humanities read scenarios in which the SpHi comparison person also was female and a member of the humanities department, whereas the SpLo and AvLo comparison people were male and enrolled in other departments of the university. To support the best possible identification with the protagonist, scenarios were written in second-person narrative (e.g., “You are a student in your last semester at the Department of Humanities”; “Your grades are only average”).

**Fig. 1.**
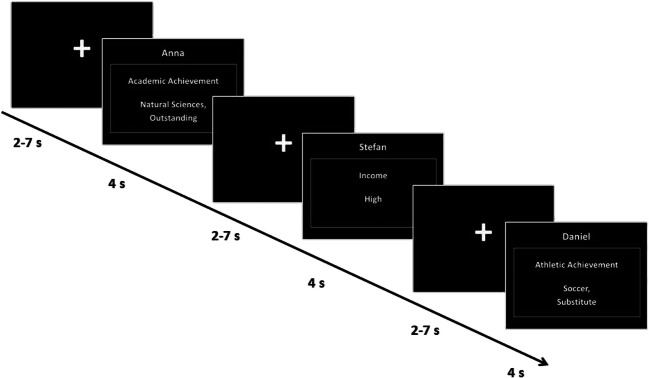
Schematic depiction of stimuli and scanner task. At the beginning of each trial, a fixation cross was presented for 2-7 s, followed by the presentation of the stimuli for 4 s. The top line indicates the comparison person; the middle line depicts the comparison domain (like performance in an important job interview, prestige of current workplace or level of income), whereas the bottom line displays the achievement of the comparison person. We presented three different comparison persons, resulting in three different experimental conditions: SpHi = superior with high similarity; SpLo = superior with low similarity; and AvLo = average with low similarity

After reading the scenarios, participants entered the scanner and performed a passive reading task in which they were presented with short reminders of each of the comparison situations previously described in the scenarios (Figure 1). Stimuli for all three conditions were presented in a randomized, event-related manner for a duration of 4 s. For each of the three conditions (SpHi, SpLo, and AvLo), 11 events (comparison domains) were each shown 4 times resulting in a total of 132 trials. For an overview of comparison domains see Table 1. A fixation cross was projected at the center of the screen during the interstimulus interval. The fixation cross duration was jittered following a Poisson distribution (2-7 s, mean = 4 s). After the scanner task, participants completed post-scan envy ratings (see below) and were then debriefed and compensated for participation.

**Table 1:** Overview of comparison domains for all three conditions

Comparison domain	Protagonist	SpHi	SpLo	AvLo
Academic achievement	Humanities, average	Humanities, outstanding	Natural sciences, outstanding	Natural sciences, average
Athletic achievement	Soccer, substitute	Soccer, best player	Volleyball, best player	Volleyball, substitute
Job interview	Average interview, multinational renowned company	Very good interview, multinational renowned company	Very good interview, local renowned company	Average interview, small local enterprise
Workplace	Small local enterprise	Multinational renowned company	Local renowned company	Small local enterprise
Salary	Low	High	High	Low
Car	Small second hand car	Expensive sports car	Modern Hybrid car	Small second hand car
House	Small apartment	Magnificent apartment, popular quarter	Magnificent house with garden in countryside	Confined apartment, unpopular quarter
Hobby	Loves collecting retro-furniture, but not enough money	Collection of expensive retro-furniture	Photography and visiting concerts	Collection of Music CDs
Weekend	Loves traveling, but not enough money	Overseas travel	Domestic travel	Walking in the park
Dinner	At home	Luxury French restaurant	Ranking German restaurant	Instant food, at home
Popularity	Few friends, no time to make new friends	Many friends, very popular	Many friends, very popular	Few friends, not popular

To note, we slightly modified the original scenarios from Takahashi and colleagues to prevent culturally biased descriptions that were inappropriate for German participants; for example, baseball (which is a very rare sport in Germany) was replaced with soccer, and high-class European car (which are more common and therefore not of comparable status in Germany) was replaced by expensive sports car.

### Envy ratings

Participants were asked to rate how much envy they experienced while adopting the perspective of the protagonist toward each of the three comparison persons on a 7-point Likert scale (1 = not at all, 7 = very strong). As the literature provides clear evidence for brain structure differences underlying social desirability bias (Andrejević, Meshi, van den Bos, & Heekeren, 2017), as well as the social undesirability of envy, including the strong tendency to underreport personal feelings of envy (Habimana & Massé, 2000; Silver & Sabini, 2006), we created the possibility to report the experience of envy via simulation, by asking how much envy our participants experienced when taking the perspective of the protagonist. Thus, we simulated subjective envy in participants by providing them with a first-person, perspective-taking measure. With this approach, we attempted to bypass social desirability biases and expected to get more accurate envy ratings. We further decided to use a single-item rating subsumed over all eleven comparison domains for each comparison person to prevent effects of fatigue and a decrease in the degree of effort and thought that respondents invest in answering that might occur when participants rated all 33 comparison domains with regard to envy levels.

### fMRI data acquisition

Imaging was performed at the Center for Cognitive Neuroscience Berlin (CCNB) using a 3T Siemens Tim Trio scanner (Siemens Healthcare Diagnostics GmbH, Erlangen, Germany) fitted with a 12-channel head coil. The task was implemented with Presentation software (Neurobehavioral Systems, Neurobs Inc., Albany, CA; Ver. 14.8; http://www.neurobs.com), running on a Dell computer under Windows XP. Stimuli were presented via MR-compatible LCD goggles (Resonance Technology Inc., Northridge, CA). Functional images were acquired with T2*-weighted gradient echo planar imaging sequences sensitive to blood oxygenation level dependent (BOLD) contrast. A total of 37 oblique slices (3- x 3- x 3-mm voxels), parallel to the anterior-posterior commissure line, were collected per volume (flip angle, 70°; TE, 30 ms; TR, 2,000 ms; matrix, 64 × 64, field of view = 111 mm, interslice gap = 0.3 mm). High-resolution anatomic images were acquired using a T1-weighted MP-Rage sequence (176 contiguous sagittal slices, slice thickness 1 mm, matrix: 256 x 256).

### fMRI data analysis

Analysis was performed using FMRIB Software Library v5.0 (FSL) (Smith et al., 2004). Preprocessing of functional data was conducted as described in the following: non-brain tissue was removed using a mesh deformation approach (Smith, 2002); slice-time correction was performed and MCFLIRT motion correction tool was applied using rigid body registration to the central volume (Jenkinson, Bannister, Brady, & Smith, 2002); Gaussian spatial smoothing was applied with a full-width half-maximum of 6 mm and high-pass temporal filtering was applied with a cutoff of 100 s. After preprocessing, first-level single subject analyses were conducted to estimate BOLD responses following a general linear model (GLM) approach with the following three regressors:

- R1. When participants were presented with information about the SpHi comparison person
- R2. When participants were presented with information about the SpLo comparison person
- R3. When participants were presented with information about the AvLo comparison person.

After computing individual contrast images, a group-level analysis, using voxel-wise one-sample *t*-tests, was performed. In order to investigate the neuronal substrates relevant for the processing of envy we performed three whole-brain contrasts: SpHi > AvLo; SpHi > SpLo; and SpLo > AvLo. For these contrasts, Z-statistic images were thresholded with default FSL cluster correction for multiple comparisons with a minimum Z-score set at 2.3 and a significance level set at *p* < 0.05.

To investigate individual differences in envy processing in the brain, parameter estimates for each participant were extracted from significant clusters of voxels in the SpHi > AvLo contrast. These regions were the left superior frontal gyrus (peak MNI coordinates −10, 40, 54; max Z = 3.87), the right angular gyrus (46, -54, 40; max Z = 4.44) and in the precuneus (−12, −60, 44; max Z = 3.34). Parameter estimates from the SpHi > AvLo contrast were then correlated with the corresponding, calculated participant envy ratings. This was the difference in envy ratings between the SpHi and AvLo conditions (Rating Difference = SpHi Envy Rating – AvLo Envy Rating).

### Functional connectivity analysis

To assess envy related functional connectivity, we performed psychophysiological interaction (PPI) analyses (Friston et al., 1997). The model was estimated in three steps (O’Reilly, Woolrich, Behrens, Smith, & Johansen-Berg, 2012). First, we identified the group peak responses of the SpHi > AvLo contrast. These were in the three significant clusters mentioned above in the left superior frontal gyrus, right angular gyrus and precuneus (see above for coordinates). We created spheres (5-mm radius) at these locations. Second, we extracted individual average time-series of BOLD signal within the seed regions. Third, for each participant, we estimated a GLM of the BOLD responses with the following three regressors:

- R1. A psychological regressor denoting the main effect of task, convolved with a double-gamma HRF
- R2. A physiological regressor denoting the activation time course of the seed region
- R3. A PPI regressor denoting the element-by-element product of the previous two (i.e., the PPI term).

Voxels exhibiting a significant task-dependent increase in coupling (positive or negative) with the seed region were identified by computing a whole-brain t-contrast on the third PPI regressor. Individual contrast images were entered in the group-level, mixed-effects analysis, using voxel-wise, one-sample t-tests. To examine individual differences in envy, we entered envy ratings (Rating Difference = SpHi Envy Rating – AvLo Envy Rating) as a covariate in the group-level analysis. For PPI analyses, whole-brain Z-statistic images were thresholded at z > 2.3 and cluster corrected to *p* < 0.05.

## Results

### Envy ratings

**Fig. 2.**
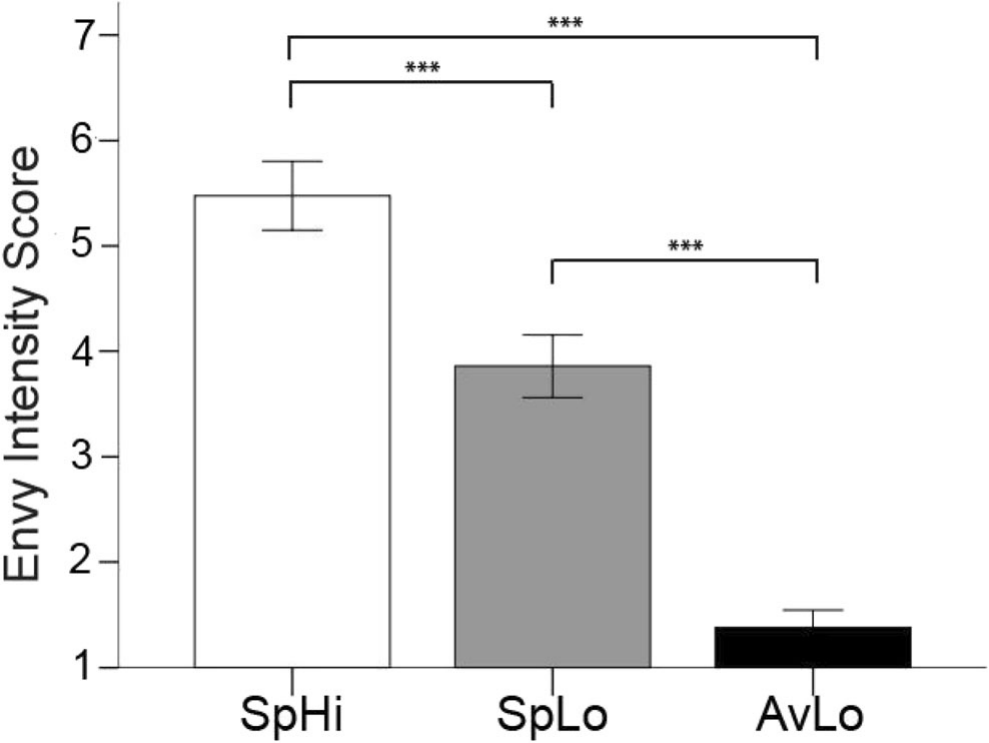
Post-experimental envy ratings for all three conditions. Significant differences between all conditions indicated by asterisk. Error bars represent ±1 SEM

Repeated measures analysis of variance of post-experiment envy ratings revealed significant differences between conditions (F(1.876, 37.53) = 72.43, *p* < 0.001; Figure 2). Subsequent post-hoc testing (dependent t-tests) revealed that the envy evoked by the SpHi comparison (*M =* 5.48, *SD =* 1.50) compared with the SpLo comparison person (*M =* 3.86, *SD =* 1.35) differed significantly *t*(20) = 4,949, *p* < 0.0001. Additionally, envy evoked by the SpHi comparison person compared to the AvLo comparison person (*M =* 1.38, *SD =* 0.74) differed significantly *t*(20) = 10.68, *p* < 0.0001. Finally, there was a significant difference between the envy evoked by the SpLo comparison person compared to the AvLo comparison person *t*(20) = 7.902, *p* < 0.0001. These results indicate that the experimental manipulation, which aimed at creating three distinct conditions across which reported experience of envy declines, was successful.

### Neuroimaging results

**Fig. 3.**
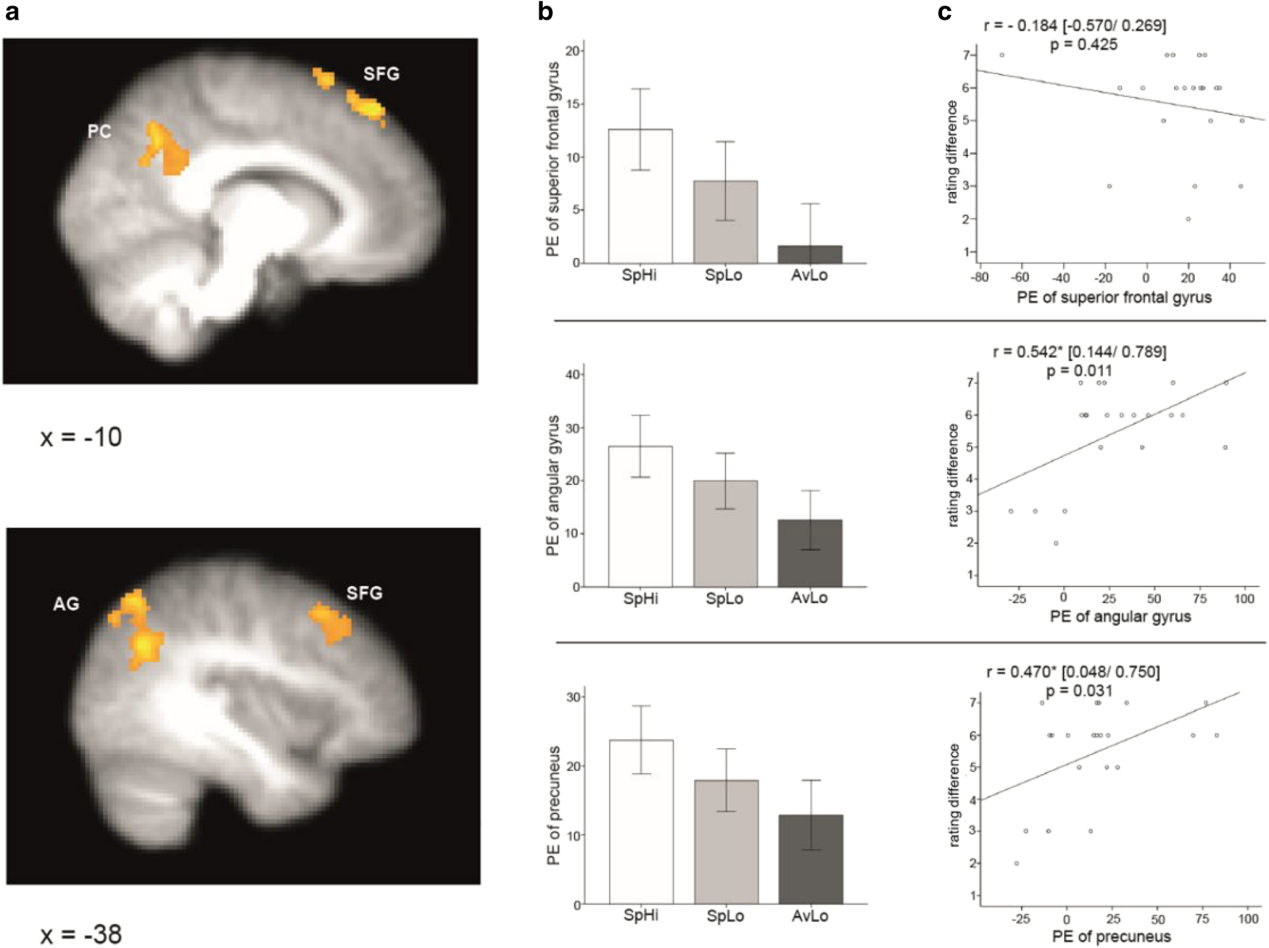
Envy recruits the superior frontal gyrus, angular gyrus and precuneus. (**a**) Brain regions demonstrating higher activation in the SpHi condition compared to the AvLo condition (SpHi > AvLo contrast). (**b**) Parameter estimates of SpHi, SpLo and AvLo conditions. (**c**) Correlations between parameter estimates from the SpHi > AvLo contrast and envy ratings (Rating Difference = SpHi Envy Rating – AvLo Envy Rating). Activation in the left superior frontal gyrus did not significantly correlate with envy scores. Activation in both the angular gyrus and precuneus positively correlates with envy rating scores. BOLD activation thresholded at Z > 2.3, *p* < 0.05, cluster corrected. SFG = superior frontal gyrus; AG = angular gyrus; PC = precuneus; PE = parameter estimate

To examine the brain’s response to envy evoking stimuli, we performed the following three contrasts: SpHi > AvLo; SpLo > AvLo; and SpHi > SpLo. For the SpHi > AvLo contrast, we found significant changes in BOLD signal in the left superior frontal gyrus (peak MNI coordinates: −10, 40, 54; max Z = 3.87; *p* < 0.05, cluster corrected), the right angular gyrus (46, −54, 40; max Z = 4.44) and the precuneus (−12, −60, 44; max Z = 3.34; Figure 3; Table 1). For the other two contrasts, SpLo > AvLo and SpHi > SpLo, no significant changes in BOLD signal that survived correction for multiple comparisons were revealed.

To examine individual differences in envy, we extracted parameter estimates from the peak voxel of the significant clusters revealed in the SpHi > AvLo contrast and performed correlation analyses with the difference in envy ratings between conditions (Rating Difference = SpHi Envy Rating – AvLo Envy Rating). These analyses revealed a significant positive association in the right angular gyrus (Pearson’s r = 0.542, 95% confidence intervals [CI] = 0.144/0.789, *p* = 0.011), and the precuneus (Pearson’s r = 0.470, 95% CI = 0.048/0.750, *p* = 0.031). Parameter estimates in the left superior frontal gyrus did not significantly correlate with the difference in envy scores (Pearson’s r = −0.184, 95% CI = −0.570/0.269, *p* = 0.425).

### Functional connectivity results

**Fig. 4.**
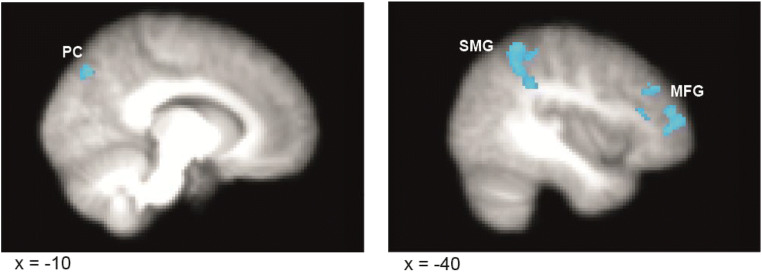
Functional connectivity of the left superior frontal gyrus exhibits an inverse relationship with envy ratings across participants. PPI analysis of the SpHi > AvLo contrast revealed significant functional connectivity between the seed region in the left superior frontal gyrus and both the supramarginal gyrus and precuneus with respect to individual envy ratings (Rating Difference = SpHi Envy Rating – AvLo Envy Rating). This connectivity exhibited an inverse relationship to the envy ratings; the greater the connectivity difference between SpHi and AvLo conditions, the smaller the envy rating difference between SpHi and AvLo conditions across participants**.** BOLD activation thresholded at Z > 2.3, *p* < 0.05, cluster corrected. PC = precuneus; MFG = middle frontal gyrus; SMG = supramarginal gyrus

To examine the functional connectivity of brain regions involved in simulated envy processing, we performed PPI analyses using seeds located at activation peaks revealed in the SpHi > AvLo contrast. These were the left superior frontal gyrus (−10, 40, 54), right angular gyrus (46, −54, 40), and left precuneus (−12, -60, 44). We wanted to test the degree that functional connectivity of these regions was related to an individual’s degree of reported envy. To this end, we entered envy ratings (Rating Difference = SpHi Envy Rating – AvLo Envy Rating) as a covariate in the group-level PPI analyses. Only the seed region in the left superior frontal gyrus yielded significant results which survived correction for multiple comparisons (Z > 2.3; *p* < 0.05). Our analyses revealed an inverse relationship between functional connectivity of the left superior frontal gyrus to both the supramarginal gyrus (peak MNI coordinates: −40, −50, 58; max Z = 3.24) and the precuneus (−10, −74, 42; max Z = 3.88) with respect to individual differences in envy ratings (see Figure 4 and Table 2 for a complete list of results). Therefore, across participants in the SpHi > AvLo contrast, the greater the functional connectivity between the left superior frontal gyrus and the two above-mentioned regions, the smaller the difference between envy in the SpHi condition and the AvLo condition (Table 3).

**Table 2:** Significant activation clusters for envy

Region	MNI coordinates			Cluster size	Peak Z
	x	y	z		
SpHi > Avlo					
L Superior frontal gyrus	-10	40	54	1425	3.87
R Angular gyrus	46	-54	40	1137	4.44
R Cerebellum	46	-68	-38	781	3.65
L Precuneus	-12	-60	44	525	3.34
SpHi > SpLo					
None					
SpLo > AvLo					
None					

*Z* > 2.3, *p* < 0.05, cluster corrected. L, left; R, right.

**Table 3:** Significant activation clusters for PPI analyses of the SpHi > AvLo contrast with individual differences in envy (SpHi rating – AvLo rating)

Region	MNI coordinates			Cluster size	Peak Z
	x	y	z		
L Superior frontal gyrus					
R Lingual gyrus	22	−62	−12	1052	3.84
L Supramarginal gyrus	−40	−50	58	508	3.24
L Middle frontal gyrus	−42	38	32	502	3.49
L Precuneus	−10	−74	42	451	3.88
R Angular gyrus					
None					
Precuneus					
None					

*Z* > 2.3, *p* < 0.05, cluster corrected. L, left; R, right.

## Discussion

We presented participants in the scanner with perspective-taking scenarios involving social comparisons to three target individuals who varied in achievements and similarity of personality. After the scanner task, participants reported their subjective experience of envy from the perspective of the protagonist in each overall situation. Our experimental manipulation successfully induced the simulated experience of envy, with participants reporting the greatest experience of envy when conducting an upward social comparison to a similar person (SpHi condition). Our whole-brain neuroimaging analysis revealed three regions that were significantly more active in the SpHi condition than the AvLo condition: the right angular gyrus, precuneus, and left superior frontal gyrus. No other contrast revealed significant results. Importantly, the SpHi-AvLo contrast is the expected contrast to induce the greatest experience of envy, as superiority and similarity of self-relevant comparison domains are the necessary preconditions for inducing envy (Salovey & Rodin, 1984; Schaubroeck & Lam, 2004; Smith et al., 1999). We further assessed whether activation in these regions correlated with reported envy across participants, with an individual differences analysis revealing significant correlations in the right angular gyrus and precuneus.

The results of our whole brain analysis indicate that the dorsomedial PFC, which includes the medial side of the superior frontal gyrus, is involved in the simulated experience of envy. To date, numerous regions of the PFC have shown activation during envy evoking social comparison situations (Harris & Fiske, 2007; Santamaría-García et al., 2017; Shamay-Tsoory et al., 2007; Takahashi et al., 2009; Xiang et al., 2016). Specifically, the dorsomedial PFC plays a wide role in social cognition and emotional processing (Dixon, Thiruchselvam, Todd, & Christoff, 2017), including adopting the perspective of others (Andrews-Hanna, Saxe, & Yarkoni, 2014; Fletcher et al., 1995; Gallagher & Frith, 2003), processing representations of the self and others (D’Argembeau et al., 2005; Denny, Kober, Wager, & Ochsner, 2012; Mitchell, Neil Macrae, & Banaji, 2005; Murray, Schaer, & Debbané, 2012; Richell et al., 2003; Yaoi, Osaka, & Osaka, 2009) and reappraisal of negative emotions (Etkin, Egner, & Kalisch, 2011). In particular, the dorsomedial PFC has been hypothesized to appraise the mental states and traits of others in relation to outcomes that affect one's own well-being (Dixon et al., 2017). For example, dorsomedial PFC activation has been observed when participants monitor and exploit an opponent's future actions for monetary gain (Hampton, Bossaerts, & O’Doherty, 2008) and when participants track a confederate's trustworthiness to maximize earnings (Behrens, Hunt, Woolrich, & Rushworth, 2008). As discussed in the *Introduction*, the primary evolutionary function of envy as a negative emotion is the promotion of behavior to gain an advantage possessed by a self-similar competitor who endangers the envier’s social standing. Thus, both cognitive and affective appraisal of how a target individual will influence the participant’s status and advantage is to be expected. On this basis, the activation we observed during the SpHi-AvLo contrast supports and extends this hypothesized function of the dorsomedial PFC, with the appraisal of the competitive risk posed to the self by a similar, superior target (in contrast to an average, dissimilar target) anticipated during the experience of envy.

In addition to the dorsomedial PFC, activation of the right angular gyrus and precuneus was significant during the SpHi-AvLo contrast. Both the angular gyrus and precuneus receive connections from numerous regions across the brain and have correspondingly been implicated in various social and cognitive processes (Cavanna & Trimble, 2006; Seghier, 2013). A wealth of evidence demonstrates that the precuneus and angular gyrus are often recruited together during representations of both the self and others, with significant overlap between self and other processing at the neural level (Legrand & Ruby, 2009). Interestingly, our individual differences analysis revealed that activation in both the right angular gyrus and precuneus positively correlated with reported envy across participants. We interpret this result as reflecting the right angular gyrus’ and precuneus’ role in the participants evaluating a representation of the protagonist-as-self compared with that of a similar, superior target individual. In line with this interpretation, Legrand and Ruby (2009) have proposed that the precuneus and angular gyrus are key nodes in a distributed network involved in inferential processing and memory recall (termed the “Evaluative-“ or “E-Network”), which is recruited during self-other evaluative processes. Previous findings have demonstrated that increased right temporoparietal junction activation is observed during incongruent social judgments involving similar (familiar social background) compared to dissimilar (foreign social background) comparisons (Saxe & Wexler, 2005). In addition, Fliessbach et al. (2007) have shown that larger reward discrepancies between experimental partners is associated with greater activity in the angular gyrus and precuneus. Importantly, an incongruence between the envied individual’s similarity to the envier, while possessing a superior trait, is considered a defining feature of envy. We thus interpret the results of our individual differences analysis as follows: The extent to which a participant experienced incongruence between the protagonist’s similarity to the target and the target’s superior trait, the greater the social comparative processing required, which is reflected in the corresponding precuneus and right angular gyrus activation. The outcome of this social comparative processing is likely exchanged by the precuneus and right angular gyrus to functionally and anatomically connected brain regions, including regions of the PFC previously implicated in the appraisal of emotional stimuli (Amting, Greening, & Mitchell, 2010; Campbell-Sills et al., 2011; Etkin et al., 2011; Phan, Wager, Taylor, & Liberzon, 2002; Viviani et al., 2010) and implicated in the experience of envy (Harris & Fiske, 2007; Santamaría-García et al., 2017; Shamay-Tsoory et al., 2007; Takahashi et al., 2009; Xiang et al., 2016). In turn, this information exchange may modulate affective states during upward social comparisons, with greater perceived incongruence between the protagonist-as-self and a superior, similar target producing an increase in simulated envy. Conflicting somewhat with our interpretation, Santamaría-García et al. (2017) found that the precuneus and angular gyrus are disrupted in patients with behavioral variant frontotemporal dementia, with reduced gray matter volume in these regions negatively associated with the experience of envy. However, given the widespread impact behavioral variant frontotemporal dementia has across the brain and especially on the frontal lobe (Seelaar, Rohrer, Pijnenburg, Fox, & Van Swieten, 2011), their results may not reflect a specific impact on envy processing but rather a broader disruption of the cognitive networks involved in social cognition and emotional processing. Based on this, we believe that the above interpretation of our individual differences analysis holds for healthy participants.

Taken together, the dorsomedial PFC, right angular gyrus, and precuneus subserve a range of functions associated with social decision-making, mentalizing ability, and representations of the self and others (Cavanna & Trimble, 2006; Lieberman, 2007). These regions also are known to be functionally and structurally connected (Cavanna & Trimble, 2006; Seghier, 2013) and while they show activation individually during non-social tasks (Seghier, 2013; Simmonds, Pekar, & Mostofsky, 2008), co-activation is rarely observed except in social scenarios requiring mentalizing, social appraisal, external-agency attribution, and self-representation (Cavanna & Trimble, 2006; Legrand & Ruby, 2009; Schurz, Radua, Aichhorn, Richlan, & Perner, 2014; Seghier, 2013; Sperduti, Delaveau, Fossati, & Nadel, 2011). Our results thus extend these previous findings by demonstrating that co-activation also occurs during the comparison of an adopted protagonist-as-self perspective to a superior, similar target individual (in contrast to a lower, average target individual), while producing reports of envy.

Following these analyses, we turned to our primary research goal and investigated the functional connectivity of the identified regions. Our PPI analyses revealed an inverse relationship in the connectivity of the left superior frontal gyrus (i.e., the dorsomedial PFC) to both the right supramarginal gyrus and the precuneus with respect to reported envy ratings across participants. No significant connectivity was revealed by our PPI analyses with seeds in the right angular gyrus and precuneus. Our results demonstrate that the greater the functional connectivity the dorsomedial PFC shared with the right supramarginal gyrus and precuneus, the less simulated envy a participant reported experiencing.

The dorsomedial PFC has diverse connectivity throughout the brain. Weak anatomical connections have been found between the dorsomedial PFC and regions involved in sensation and memory (Barbas, Ghashghaei, Dombrowski, & Rempel-Clower, 1999; Ray & Zald, 2012), while stronger anatomical and functional connections are made between the dorsomedial PFC and the temporoparietal junction, precuneus, and temporal poles, with co-activation of these regions often observed in tasks requiring mentalizing, self-other evaluative processing, and default-mode network activation (Andrews-Hanna et al., 2014; Barbas et al., 1999; Gusnard, Akbudak, Shulman, & Raichle, 2001; Kestemont et al., 2015; Legrand & Ruby, 2009; Mar, 2011; Meshi et al., 2016; Petrides & Pandya, 2007; Santiesteban, Banissy, Catmur, & Bird, 2012; Silani, Lamm, Ruff, & Singer, 2013; Van Overwalle, 2009; Zahn et al., 2007). Based on these functional and anatomical connections it has been suggested that the dorsomedial PFC is a major node within a distributed network broadly associated with social cognitive processing (Schurz et al., 2014). Additional interconnections extend from the dorsomedial PFC to other regions of the PFC, including the lateral orbitofrontal cortex, broadly implicated in emotional processing (Etkin et al., 2011; Phan et al., 2002), the rostromedial PFC, involved in emotion regulation strategies (Amting et al., 2010; Campbell-Sills et al., 2011; Morawetz, Bode, Baudewig, Kirilina, & Heekeren, 2016; Morawetz, Bode, Derntl, & Heekeren, 2017; Viviani et al., 2010), and the pregenual anterior cingulate cortex (Barbas et al., 1999; Fatfouta, Meshi, Merkl, & Heekeren, 2018; Öngür & Price, 2000; Petrides & Pandya, 2007)

A possible explanation for our PPI result relates to the dorsomedial PFC’s role in emotional reappraisal. In addition to involvement in social cognition, previous findings have shown the dorsomedial PFC also plays a key role in the cognitive reappraisal of emotionally salient stimuli (particularly negative emotions), with the magnitude, duration and quality of the emotion a direct result of the reappraisal process (Etkin et al., 2011; Gusnard et al., 2001; Heinzel et al., 2005; Lane, Fink, Chau, & Dolan, 1997; Morawetz et al., 2016, 2017; Northoff et al., 2004). Taken more broadly, the dorsomedial PFC’s role in the cognitive reappraisal of emotions has been argued to result from domain-general processes involved in monitoring and evaluating changing emotional states (Buhle et al., 2014). This domain general interpretation of emotion reappraisal by the dorsomedial PFC is further consistent with the regions hypothesized role in the appraisal of another’s mental states and traits with respect to facilitating one’s advantage (Dixon et al., 2017). Indeed, the appraisal of how another’s mental state influences one’s well-being is a central dimension of emotional appraisal in several psychological models of emotion (Brosch & Sander, 2013; Dixon et al., 2017; Lazarus & Smith, 1988; Scherer, 2001). As discussed above, the precuneus and right temporoparietal junction (to which both the angular and supramarginal gyri belong) are broadly involved in social cognition and self-other inferential information (Legrand & Ruby, 2009). Based on these results, we propose that the functional connectivity observed between the left dorsomedial PFC and right supramarginal gyrus/precuneus reflects the emotional reappraisal of upward social comparisons during the simulation of self-other evaluative processing. In other words, information from the right precuneus/supramarginal gyrus about the adopted protagonist-as-self, compared to a target individual, is exchanged with the dorsomedial PFC, which in turn reappraises the emotional value of the information with respect to how the target individual influences the protagonist-as-self’s well-being or advantage. In turn, increased functional connectivity between the dorsomedial PFC and right supramarginal gyrus/precuneus allows for greater emotional reappraisal of self-other evaluative information that could potentially elicit a negative emotion, in this case envy. Such emotional appraisal processes by the dorsomedial PFC may ultimately lead to reduced reports of the envy by the participants. Thus, our results extend previous findings on the dorsomedial PFC’s role in emotion reappraisal by showing that greater functional connectivity between the left dorsomedial PFC and right supramarginal gyrus/precuneus correlates with a reduction in reports of the negative emotion of envy.

Although our study design was modeled after Takahashi et al. (2009), we did not replicate their findings. Specifically, our experiment did not reveal dorsal ACC during the SpHi-Avlo contrast where participants compared themselves to a similar, superior target individual as opposed to an average, low similarity target individual. There are a couple possible explanations for this. First, Takahashi et al. recruited Japanese participants, while our participants were German university students. Research from the field of cultural neuroscience suggests that cultural variation impacts brain processes involved in several cognitive domains, including self-representation, emotion, and motivation (Ames & Fiske, 2010; Chiao et al., 2009; Han et al., 2013; Kitayama & Park, 2010; Korn et al., 2014). It is thus possible that the processing of social comparisons and envy is differentially shaped by cultural background. Indeed, initial evidence for culturally-based differential shaping of neural processes underlying social comparison was recently presented (Kang, Lee, Choi, & Kim, 2013). Second, Takahashi et al. did not correct for multiple comparisons for their a priori hypothesized regions, while all our results were corrected for multiple comparisons. Against this background of differences in sample selection and statistical analysis, we did not replicate the results of the initial study. Nevertheless, as described above, our results are in line with other previous studies on social comparison and self-other cognitive processes. In addition to not finding AAC activity, we did not observe any activity in the ventral striatum. This is of note, as previous studies have observed ventral striatum activity during investigations into social comparison and envy (Takahashi et al., 2009; Dvash et al., 2010). This lack of neural activity is likely due to our paradigm focusing specifically on the negative emotion of envy and not the pleasurable emotion of schadenfreude.

The present study also includes a limitation that warrants mention. Envy was not evoked via the participant’s direct subjective comparison with the target individual. Instead, the participants took the perspective of a protagonist when performing social comparisons. Removing the participants’ own direct involvement in the social comparisons potentially reduced the affective experience of envy. Furthermore, it is probable that task-specific mentalizing processes influenced the observed neural activity, resulting in the findings being best interpreted in the context of simulated subjective, rather than direct subjective envy. We chose our design to avoid envy being underreported, a common issue in such research due to displays of envy often being viewed as socially undesirable (Habimana & Massé, 2000; Silver & Sabini, 2006). Replicating our results with a design able to evoke subjective feelings of envy while avoiding issues with underreporting may improve upon our findings.

When thinking about future research, one can consider that recent research has revealed profound negative effects of envy on subjective well-being and social interactions. For example, dispositional envy is correlated with higher levels of depression and neuroticism (Smith et al., 1999), while also hindering cooperation (Parks et al., 2002), affecting group performance negatively (Vecchio, 2005), and promoting irrational decision-making (Beckman, Formby, James Smith, & Zheng, 2002; Zizzo & Oswald, 2016). Therefore, our findings relating neural activation associated with emotional reappraisal to reduced self-reports of envy has the potential to be relevant for both educational and clinical research in the future.

## Conclusions

Our study found an inverse relationship in the functional connectivity of the left superior frontal gyrus to both the supramarginal gyrus and the precuneus with respect to self-reported envy ratings across participants. This finding thus extends our present knowledge of the superior frontal gyrus’ role in the reappraisal of negative emotions and in modulating the experience of the negative social emotion of envy.

### Open practices statement

None of the data or materials for the experiments reported here is available, and none of the experiments was preregistered.
